# Psychological factors affecting potential users’ intention to use autonomous vehicles

**DOI:** 10.1371/journal.pone.0282915

**Published:** 2023-03-16

**Authors:** Tianyang Huang

**Affiliations:** School of Mechanical Engineering, Guangdong Ocean University, Zhanjiang, China; Babes-Bolyai University, Cluj-Napoca, ROMANIA

## Abstract

As a currently emerging technology and an emerging intelligent mode of transport, autonomous vehicles (AVs) with lots of potential advantages need attention in terms of acceptability of their users. This research incorporates three psychological factors of perceived trust, perceived value, and perceived enjoyment into the technology acceptance model, and explores the influence of these factors on the potential use intention of AVs users. In this study, the questionnaire data from 232 participants were analysed, and the structural equation model test study model was adopted, and nine hypotheses proposed in this study were verified. The results show that perceived enjoyment, perceived trust, perceived usefulness, and attitude have a direct positive impact on users’ usage intentions. Perceived value, perceived usefulness, and perceived ease of use have a direct positive impact on user attitudes. In addition, perceived ease of use has also been shown to directly affect perceived usefulness. This study constructs and demonstrates a model of autonomous vehicle acceptance. This model can be used for user acceptance research of unmanned vehicles. The research expands the theory of technology acceptance model and its applicable fields, and enriches the theory of user research on unmanned vehicles. This study provides predictors of AVs acceptance for AVs designers, automakers, automotive policy makers, and related practitioners. Help them make actionable autonomous vehicle-related decisions to promote high-acceptance autonomous vehicle design and user intent for autonomous vehicles.

## Introduction

More innovative development schemes have been worked out by virtue of the innovation of future urban traffic facilities along with the development of artificial intelligence technology, and the automated vehicle technology which much attention has been paid to in recent years is a typical representative among them. Autonomous vehicle (AVs), also called auto-driving vehicle, refers to a motor vehicle that can perceive the road environment and be automatically navigated when not being under the driving control of a human driver [1, 2]. Driverless cars have attracted the attention of various countries and regions in different periods. For example, the CyberCars project launched by the European Union in 2001 uses laser scanning technology to realize the functions of obstacle detection, vehicle tracking and autonomous navigation of intelligent vehicles [3]. China’s Baidu smart car passed the approval of the California Department of Motor Vehicles in 2016, and obtained a permit for road testing of unmanned vehicles. This is an important event in China’s smart car technology [4]. In 2021, Beijing launched China’s first commercial autonomous driving travel service pilot project [5]. At the same time, traffic legislation [6], parking policy [7], road competitiveness [8], vehicle intelligence technology and algorithm (such as remote sensing data fusion technology, motion planning and target recognition algorithm, collision avoidance technology) [9, 10] and so on have gradually attracted the attention of researchers. Although self-driving cars have taken some time to develop, they may not become popular in the market until 2040 at the earliest, or as late as 2060 [11, 12]. In advanced economies, by 2050, highways will be almost unmanned, and the global market for self-driving cars is expected to reach about 400 billion U.S. dollars [13]. It can be said that with the progress of such technologies as artificial intelligence, advanced vehicles, etc., AVs have received much attention in recent years [14–16], which provide a more safe and sustainable mode of transport for tourism [17, 18]. These AI-powered AVs are gradually becoming part of our daily lives [17]. Due to the subversion of AVs, people’s travel methods and travel distances have changed [17], reshaping the mobility and sustainability of travel [19].

AVs have many advantages. For example, because AVs can drive automatically, the driver does not need to pay complete attention to the road, but handle his personal affairs during the ride, greatly improving the use efficiency of the driving time [20]. AVs’ users can reduce their driving time and improve their driving efficiency through such functions and services as reservation, effective use of lane, route optimization, etc. [21, 22]. AVs can prevent the humans from getting tired, distracted, etc. during vehicle driving, possibly greatly decrease unnecessary traffic accidents [21], and can provide personal mobility service for juveniles and disabled people [21]. AVs are helpful for optimizing traffic flow [16] and reducing traffic accidents [23]. On the whole, AVs are advantageous in respect of safety improvement, energy saving, emission reduction and enhancement of transport efficiency, etc. [24–26]. Despite the many benefits of AVs, user adoption of AVs appears to be lagging far behind expectations [27]. For example, in the study of Menon et al. [28], it was found that more than half (61.5%) of American drivers were unwilling to take AVs [28], and their willingness to use or purchase AVs was very low. Therefore, it is quite important to learn personal users’ attitude towards automated vehicles, because it is a key factor in the individuals’ future technological demand as well as the organizations’ future governance policy and investment in future traffic infrastructure [28], and the acceptability to AVs can be promoted only after the factors affecting the intention to use AVs are learned [29].

In order to predict and explain user behavior and clarify the relationship between users’ attitudes toward technology and technical behavior, previous studies have borrowed the user technology acceptance model (TAM) [30, 31]. For example, Wu et al. [32] conducted a research on the public acceptance of self-driving buses based on the technology acceptance model. The results show that attitudes positively affect users’ behavioral intentions, and trust and perceived usefulness variables positively affect users’ attitudes. This study extends the technology acceptance model. Park et al. [14] added social influence and facilitating condition to the technology acceptance model, and also explored the moderating effects of demographic variables such as age, family size and education level. The results show that three variables, social influence, convenience and perceived usefulness, are important factors affecting AV users’ usage intention. At the same time, demographic variables play a moderating role in the effects of perceived usefulness and social influence on AV use intention. Although relevant studies have determined the impact factors of users’ acceptance and use of AVs, the dimensions and relationships of technology acceptance model in some studies are still not very clear [33], in some studies, the relationship between perceived ease of use and perceived usefulness and intention of use is not significant [34, 35], which is different from what were found in the TAM. Just as what were pointed out in the past studies [36, 37], the predictive ability of the TAM can still be enhanced by extension, although it is a theoretical model that has been widely applied. Although the technology acceptance model is an excellent predictive model of technology acceptance, it does not include some other significant belief variables [38], and it needs to be extended to the environment of autonomous vehicles. Thus, this study tries to increase new dimensions based on the TAM model, in order to further explain the acceptability of AVs.

It is extremely important to carry out research on users’ psychological factors [39]. Although research on user behavior of driverless cars has gradually attracted attention, the psychological factors were focused on only in a small number of previous studies [35, 40, 41]. Accordingly, the studies on such psychological factors as perceived trust, perceived value and perceived enjoyment are slightly insufficient, although the study on the behaviors of AV users has gradually received attention. Verberne et al. [41] pointed out that the dimension of perceived trust, as an important psychological factor explaining acceptability to AVs, can have a positive influence on the adoption behavior of automated information technology users [41, 42]. The automated driving technology, as an automated system, has a relationship with humans that is under the action of trust [29]. Just as Choi et al. [35] put it, users’ trust, as a key factor affecting users’ intention to use AVs, can enhance users’ intention to use AV technology [34, 43, 44]. Meanwhile, Users’ perceived value is the results of their examining and weighing product capabilities and assessing product efficacies based on the integral technology gain and loss [45], and as a subjective evaluation indicator, it is used a useful tool for analyzing and studying the relationship between behavioral intentions and behavioral decision making [46]. That is to say, the psychological activity about such perceived value can affect the user’s behavioral intention and finally affect his actual behavior. This has been demonstrated in previous studies, for example, Ström et al. [47] pointed out that perceived value has a positive influence on consumers’ behavior of changing purchase channels. Yuen et al. [48] pointed out that perceived value has a positive impact on users’ attitudes towards technology. In addition, Li et al. [46] also pointed out that different customers’ perception of the value of the same product can differ due to different channels, in the multi-channel clothes purchase environment and market. All of these indicate to a certain extent that the differences in situation and field can affect the users’ perceiving value. Thus, the intention to use automated vehicles and the influences of perceived value deserve our discussion. In Steg’s opinion [49], driving, a composite behavior, is risky, exciting and pleasant. According to psychological theories, behavioral motivations consist of intrinsic and extrinsic motivations [50, 51], and enjoyment is a typical intrinsic motivation [52]. Those preferring to obtain enjoyment from excitement and risks and seeking after novel experience are willing to accept use of new technology systems early [53, 54], because they can more feel the novel attraction of technology to them by early experiencing technology. Just as Holbrook et al. [55] put it, consumers may choose to use a new technology, due to their focus on the fun and even novelty in it. However, Haboucha et al. [56] pointed out that those having their own driving habits desire to control vehicles, and thus are impossibly willing to use AVs, because AVs can deprive them of their fun in driving experience. Therefore, the efficacy of perceived enjoyment on use of AVs still needs to further studied [57].

Although there are many current studies investigating users’ behavioral intentions for autonomous vehicles, they are still not comprehensive [39]. The current research on the behavioral intention of autonomous vehicles has problems such as the use motivation is not unified [58]. For the study on the intention to use AVs, it is necessary to discuss from the perspective of users’ psychological factors [33], in order to enhance the knowledge about use of AVs. Through a theoretical analysis on psychological factors, the public’s acceptance and use of AVs can be more fully understood. However, the work related to studying the users’ acceptance of AVs is quite limited, and the exploration into the relevant influencing psychological factors has led to few results [59, 60]. Many current studies focus on demographic variables [14, 61], and currently there are many studies on tourists [17, 18] and drivers with driving experience [34, 62], and there is a lack of student groups as samples. However, the only individual research [63] with college students as samples is from the designer’s perspective. The current college students are an important group of potential users of unmanned vehicles in the future. It may be helpful to understand the influencing factors of their AVs use intentions, And for the subsequent wider commercialization of AVs. Thus, this study aims at discussing the acceptance and use by AV users from the perspective of psychological factors, that is to say, the three psychological factors, i.e., perceived trust, perceived value and perceived enjoyment, are included in the technology acceptance model, for discussing their influences on the intention of potential users such as college students of AVs. This study extends the technology acceptance model by adding three psychological factors (perceived trust, perceived value, and perceived enjoyment) that influence autonomous vehicle user intentions to the technology acceptance model. Research builds and validates autonomous vehicle acceptance model. The findings of this study could improve our understanding of the factors associated with user adoption of autonomous vehicles.

## Research hypothesis development

### Technology acceptance model

The technology acceptance model proposed by Davis [64] is based on Theory of Reasoned Action. It is a representative theoretical model for explaining and predicting the adoption of technological systems [37, 65]. The model includes variables such as behavioral intention to use, attitude, perceived usefulness, and perceived ease of use. It is widely used in technology acceptance studies [66]. In addition, in recent years, the TAM has been applied in the relevant studies on acceptance and prediction of automobile driving, e.g., Kaur et al. [67]., and Zhang et al. [68].

Users’ intention are the basic requirements for adopting a new technological system [69]. For the early study stage of a technology, BI is frequently taken as a dependent variable [70, 71]. It is impossible to test the actual use of AVs, because they have not been widely applied yet. Therefore, it is rational to take intention as a result variable. In the TAM, attitude means users’ positive or negative attitude towards technology use. If believing in that adopting a technology can lead to a good result, individuals will have a positive attitude towards adopting it. In the TAM, users’ attitude towards technological behavior, as a key factor for predicting and explaining human behaviors, positively acts on the use intention. Previous studies [72–74] also demonstrated that attitude can positively affect users’ intention to use AVs and other new innovative technologies. In Wu et al. ’s research [32] on the public acceptance of autonomous buses, it is also shown that user attitudes and user intentions have a positive impact. In addition, Wang et al. [75] collected data through online surveys and explored the antecedents that influence Chinese consumers to use electric shuttle bus service. The results showed that attitudes have the greatest impact on behavioral intentions. This study believes that the users’ intention to use AVs, an innovative technology product, should also be affected by attitude. For this purpose, we put forward:

**H1**: ATT has a positive influence on the users’ intention to use AVs.

In the TAM, perceived usefulness (PU) is defined as the degree to which the user believes that they can improve their performance by using an information technology system, and it can also be regarded as the function and advantage that can be achieved through use of technologies [70]. Perceived usefulness significantly affects users’ intention for and attitude towards using technologies [64]. Such advantages of AVs as optimization of road planning [21, 76] may also enhance the user’s intention to use them to a certain degree, and just as Lee et al. [33] put it, for AVs, the leading effect of the user’s perceived usefulness on their intention to use AVs can be considered as a significant feature of AVs. The perceived usefulness of driverless cars has a positive impact on users’ intentions [77]. In the study of Park et al. [14] who used demographic variables as key factors to explore user adoption of autonomous vehicles, they also pointed out that perceived usefulness has a positive impact on user adoption. The research of Jászberényi et al. [18] also shows that the perceived usefulness variable has a positive impact on the user’s intention to use self-driving cars for travel. Based on TAM and other theories, Stiegemeier et al. [78] conducted a study on the acceptance of in-vehicle assistance and infotainment systems for German drivers, showing that perceived usefulness affects users’ use intentions. At the same time, the positive impact of perceived usefulness on users’ usage intentions has also been confirmed in the studies of Li et al. [79] and Yan et al. [80]. Of course, the relationship between perceived usefulness and usage intention is also specified in TAM [70]. In addition, related research [81] also shows the positive and direct impact of perceived usefulness on attitudes. Based on the above studies, this study proposes that the perceived usefulness of users’ belief that using Avs will enhance their job performance may positively influence users’ attitudes and intentions to use Avs. Therefore, we assume:

**H2**: Perceived usefulness positively affects users’ attitude towards use of AVs.

**H3**: Perceived usefulness positively affects users’ intention to use AVs.

Davis et al. [70] interprets perceived ease of use as the degree to which users perceive using a particular technological system as effortless or easy. In this study, PEOU refers to the degree to which AVs can be easily used in an individual’s opinion. In the technology acceptance model, PEOU, as an important factor, positively affects the PU and the attitude of the user using the technology, and thus indirectly affects the use intention. The relevant studies on acceptance and use of AVs also demonstrated the role of perceived ease of use [35, 65]. For instance, a study of Lee et al. [33] showed that perceived ease of use affects perceived usefulness. In addition, a previous study [35] also pointed out that PEOU positively affects use intention, and the significant impact of perceived ease of use on attitudes [81]. Koul et al. [77] used Pearson correlation analysis and multiple linear regression analysis to explore the impact of driverless car technology’s perceived ease of use on usage intention. The results show that perceived ease of use has a significant positive impact on usage intention. In Jászberényi et al. ’s study [18] on the intention to adopt autonomous vehicles for tourism purposes, it was shown that perceived ease of use has a significant impact on the intention to use. In addition, the positive impact of perceived ease of use on user intentions has also been confirmed in research on mobile payment technology [82], vehicle assistance and entertainment system [78] and e-learning technology [83]. However, some previous studies [20, 84] showed that perceived ease of use has no effect on intention to use. In addition, the direct effect of perceived ease of use on perceived usefulness is also often confirmed by some studies [32, 74, 85]. This study argues that the impact of perceived ease of use on user intentions may vary across technical systems. In this study, as an innovative product, the ease of use of AVs is also extremely important, because the ease of use may also affect users’ perception of the usefulness of AVs, their attitudes, and their intentions. Therefore, based on previous research, this study put forward:

**H4**: PEOU positively affects users’ attitude towards use of AVs.

**H5**: PEOU positively affects users’ perceived usefulness about AVs.

**H6**: PEOU positively affects users’ intention to use AVs.

### Perceived trust

Lee et al. [86] defines trust as: "the attitude that an agent will help achieve an individual’s goals in a situation characterized by uncertainty and uncertainty Vulnerability ". Furthermore, Herrenkind et al. [87] interprets trust as the degree of trust in the predictability and functionality of a particular technical system. In addition to regulating the relationship between individuals, trust can adjust the relationship between humans and automation technology [35], and it is regarded as the main decisive factor for accepting and using automation technology [35, 67, 86, 88–90]. However, it is unadvisable to excessively or insufficiently trust with technology use. Parasuraman et al. [91] pointed out that insufficient trust in automated system may lead to losing the opportunity of using automated system technology, and excessive trust in it can result in misuse of automated system technology. Just as Jing et al. [92] pointed out that perceived trust was not taken seriously in the early technology acceptance model. However, in the current lack of abundant AVs acceptance research, related research also shows that trust is a significant predictor of users’ intention to use AVs [93]. For instance, Choi et al. [35] and Zhang et al. [30] pointed out that trust plays a key role in users’ accepting AVs, and the relevant studies also demonstrated the role of trust in the users’ intention to use AVs [35, 67, 92]. It has the greatest impact on the acceptance of AVs [94]. We hold that Potential users should consistently trust the emerging technology AVs, and only this can ensure that there are further attempts to use them. Therefore, we put forward:

**H7**: Perceived trust positively affects users’ intention to use AVs.

### Perceived enjoyment

Entertainment motivation is the foundation on which consumers’ consuming behavior is understood [95]. Driving fun can be construed as users’ pleasant experience and feeling related to their intrinsic motivations during automobile driving [52, 96, 97]. The relevant studies [54, 98, 99] showed that entertainment motivation is one of the important decisive factors affecting use and adoption of new technology systems. A study of Gkartzonikas et al. [100] showed that users’ intention to use AVs is under the influence of their motivations like novel demands, driving experience and enjoyment, etc. In the study of Benleulmi et al. [58] on the behavioral use intention of fully autonomous vehicles, it was also confirmed that hedonic factors have a significant positive impact on the use intention. Madigan, et al. [101] also found that enjoyment is the strongest factor predicting users’ intention to use AVs. Perceived enjoyment can positively act on users’ intention to use technology [87]. Therefore, in this study, we assume:

**H8**: Perceived enjoyment positively affects users’ intention to use AVs.

### Perceived value

During the research and development of AVs, examining and weighing users’ value is necessary, and this value is also required to be considered in the early study on use intention. Perceived value is construed as the weighing between the benefits obtained during a user’s using a product or service and the monetary cost of adopting the product or service [45]. So, the perceived value of AVs can be understood as the weighing between the monetary cost paid by users for using AVs and the benefits obtained by them during use of AVs [45, 54]. The perceived value will be considered by users as positive, when they perceive that the benefits obtained through use of technology systems are higher than the monetary cost paid by them for using the systems [102]. It is one of the key factors affecting users’ using technology, significantly affects users’ attitude to use technologies [48]. Hewapathirana [103] subdivided perceived value into quality, price, emotion, social and ecological values. The study confirmed that these five value dimensions have a positive impact on users’ attitudes towards alternative fuel vehicles, thereby affecting users’ Intended use of alternative fuel vehicles. Vinijcharoensri [104] investigated the impact of users’ perception of the value of luxury cars in Thailand on luxury attitudes. The study divides perceived value into three dimensions: social value, personal value and functional value, and they include different factors at the same time. The research shows that all factors in the dimension of functional value are significantly positively correlated with attitude, and some factors in the dimension of social value and personal value have a positive impact on attitude. For this reason, we infer that no matter how the dimensions of perceived value are divided, generally speaking, perceived value has a certain impact on users’ attitudes. After all, when users perceive the various benefits that new technologies such as driverless cars bring to daily life and social development, their attitudes should be positive. Thus, we propose this assumption:

**H9**: Perceived value positively affects AVs users’ attitude towards technology use.

**Fig 1:**
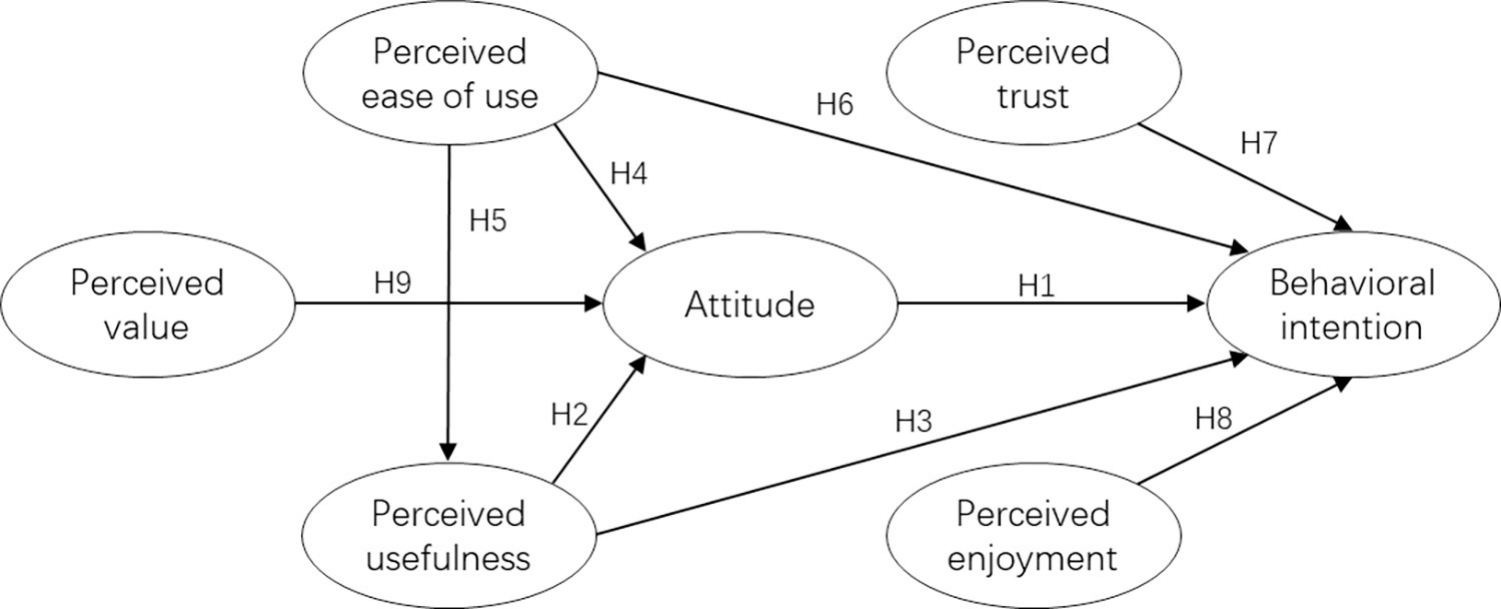
AVs technology acceptance model.

To sum up, an AVs technology acceptance model is hereby proposed for this study, as shown in Fig 1.

## Methods

### Questionnaire

In this study, a questionnaire was designed to collect data to verify our model. To guarantee the content validity of the questionnaire, all of its items were adapted from previous theoretical literature, and at the same time, two experts were invited to conduct the review to revise and adjust the wording and context of the items and enhance the users’ understanding. Later on, 23 copies of the questionnaire were pretested, and the results showed that the Cronbach’s a values of all the variables were above 0.7, manifesting that the dimensions of the questionnaire are credible, resulting in the final questionnaire. The formal questionnaire consists the scale in the first part and the personal basic information of the second part. The personal basic information consists of gender, age, whether a driver’s license has been obtained or not (with or without driver’s license), actual experience in automobile driving (with or without driving experience. that is, independent practical driving experience, non-driving experience during the driving test), knowing of AVs (having heard of it or not). In recent years, TAM has been applied in related acceptance and prediction studies of car driving, for example, the studies of Kaur et al. [67] and Zhang et al. [68]. However, although relevant studies have identified the influencing factors of user acceptance and use of AVs, the facets of TAM and their relationship are still unclear in some studies [33]. Although the technology acceptance model is an excellent technology acceptance prediction model, it does not include some other significant belief variables [38], and its application needs to be extended to the environment of autonomous vehicles. For this reason, this study is based on the science and technology acceptance model to expand, so the scale consists of the measurement items of seven dimensions of PEOU, PU, ATT, BI, PT, PE and PV. A study of Choi et al. [35] was referred to for the measurement items of the dimension of perceived trust, the four items studied by Madigan et al. [101] were referred to for the measurement items of the dimension of perceived enjoyment, and a study of Venkatesh et al. [54] was referred to for the measurement indicators of the dimension of perceived value. We prepared the measurement items of the dimensions of PEOU, PU, ATT and BI by referring to a study of Davis et al. [70]. The questionnaire of this study is shown in the Support information. The questionnaire had the 7-point Likert scale ranging from 1 (strongly disagree) to 7 (strongly agree). Before being replied to, the questionnaire provided a basic introduction to AVs, namely, “AVs refer to a motor vehicle that can sense the environment and navigate, and possibly can lead to reduction of traffic jam, automatic navigation, automatic obstacle avoidance and enhancement of travel efficiency, etc.”, for the purpose of making the participants basically learn AVs.

### Participant and data collection

The current college students are the representative group who are about to go to work. They were born and grew up in the Internet age, and they pay attention to new things. More importantly, these college students will be important potential buyers and users of driverless cars. However, currently there are many studies on tourists [17, 18] and drivers with driving experience [34, 62], and there is a lack of research on college students as samples. At the same time, the samples of many current studies [17, 34, 105, 106] are mainly European and American, while the research on Chinese users in the huge Chinese market is still insufficient. Therefore, in this study, the relevant data was collected from some students of a university in China by convenience sampling, for the purpose of being oriented towards more widely distributed potential AV users. All participants are from China, and the data was collected through the network. The study obtained the informed consent of all participants, all the participants were anonymous. The ethical approval for all experimental protocols in this study was approved by the Academic Committee of Guangdong Ocean University, and the participants verbally agreed to participate in this study under the recruitment and witness of the class teacher, and then they filled out and submitted the questionnaire voluntarily. All methods of the study were carried out in accordance with relevant guidelines and regulations. From February to March, 2021, a total of 275 copies of the questionnaire were received, with 27 of them, which were obviously defective (on which, for instance, the replies to the all the items were the same, the replies are regular, etc.), eliminated. In addition, considering that driverless cars are a new technology, in order to truly understand the intentions of users, this study also excluded the questionnaire data of 16 users who had never heard of driverless cars. Therefore, 232 questionnaires were finally included for statistical analysis in this study. The demographic characteristics are shown in Table 1. Of the repliers of the retained 232 copies, 139 (59.9%) and 93 (40.1%) were male and female respectively, aged 20.36 (SD = 1.216) on average, 97 (41.8%) had successfully obtained a driver’s license, 135 people did not get a driver’s license, 74 (31.9%) had actual experience in automobile driving, and all (n = 232) had heard of AVs.

**Table 1:** The demographic of participants (N = 232).

Items	Option	Number	Percentage (%)
Gender	Male	139	59.9
	Female	93	40.1
Small car license	Have a small car driver’s license	97	41.8
	No small car driver’s license	135	58.2
Driving experience	Have car driving experience	74	31.9
	No car driving experience	158	68.1
Heard of AVs	Have heard of AVs	232	100
Mean age	Mean = 20.36 (SD = 1.216);Minimun = 18; Maximum = 24		

### Data analysis

Partial least squares structural equation modeling is considered as more adaptable to the studies involving exploratory analysis and theoretical development [107]. It is not limited by the variable distribution form, and can handle more complex structural models, and has advantages such as multivariate analysis [108]. Therefore, in this study, Partial Least Squares-structural equation modeling were used as the method of data analysis, and BootStrap method via data Re-sampling was used to estimate the significance of path coefficients (T-value values) [109] to test the hypotheses proposed in this study. Moreover, the explanatory power of the research model was judged with *R*^2^. In addition, this study reduced the common method bias through the anonymous answers of the scale and the simple topic statement, and used the Harman single factor test to evaluate the common method bias [110]. The results of Harman’s single factor test indicated that the variance explained by the single factor maximization was 39.525%, which did not exceed the threshold of 50% suggested by Podsakoff et al. [111].

## Results

### Reliability and validity results

In structural equation modeling, the evaluation of the measurement model is the primary task to be accomplished, while the assessment of reliability and validity is the key component of the measurement model evaluation. Measuring the questionnaire reliability and validity is firstly required. According to Hair, et al. [112], the value of internal consistency (Cronbach’s alpha) should be higher than 0.7 and that of composite reliability (CR) higher than 0.7 [113]. The Cronbach’s alpha of all the dimensions of our study exceeded 0.7, that is to say, 0.735, 0.835, 0.868, 0.741, 0.906, 0.914 and 0.809 for PEOU, PU, BI, ATT, PT, PE and PV respectively. The composite reliability values of all the dimensions were higher than 0.7 (Table 2), indicating that the study had good internal consistency.

**Table 2:** Reliability and validity of variants.

Constructs	Item	Indicator Reliability	Mean	Standard Deviation	Cronbach’s Alpha	CR	AVE
ATT	1. Using driverless cars is a good idea.	0.782	4.845	1.107	0.741	0.853	0.659
	2. Using driverless cars is a wise choice.	0.862	4.810	1.090			
	3. It is very pleasant to use driverless cars.	0.789	4.784	1.097			
BI	1. I intend to use driverless cars in the future.	0.895	4.841	1.388	0.868	0.919	0.791
	2. I hope to use driverless cars in the future.	0.888	4.819	1.424			
	3. I plan to use driverless cars in the future.	0.885	4.642	1.348			
PE	1. Using driverless cars would be fun.	0.918	4.892	1.246	0.914	0.945	0.852
	2. Using driverless cars would be enjoyable.	0.930	5.034	1.159			
	3. Using driverless cars would be pleasant.	0.923	4.875	1.173			
PEOU	1. Learning to operate driverless cars would be easy for me.	0.718	4.573	1.100	0.735	0.846	0.649
	2. I would find it easy to get driverless cars to do what I want to do.	0.824	4.534	1.155			
	3. I will find driverless cars easy to use.	0.867	4.716	1.162			
PT	1. A driverless car is dependable.	0.935	4.207	1.118	0.906	0.941	0.842
	2. A driverless car is reliable.	0.935	4.250	1.105			
	3. Overall, I can trust a driverless car.	0.881	4.168	1.186			
PU	1. Using a driverless car can improve my driving efficiency.	0.814	4.517	1.263	0.835	0.890	0.669
	2. Using a driverless car can improve my travel efficiency.	0.846	4.647	1.265			
	3. Using a driverless car can reduce my driving stress.	0.792	4.996	1.331			
	4. I will find a driverless car is useful.	0.818	4.720	1.223			
PV	1. The price of driverless cars should be reasonable	0.721	5.284	1.184	0.809	0.867	0.687
	2. Driverless cars are worth the money.	0.823	5.409	1.145			
	3. Driverless cars can provide good value.	0.929	5.228	1.100			

The average variance extracted (AVE) of all the dimensions of this study were higher than 0.5, reaching the value 0.5 suggested by Fornell et al. [113] and Bagozzi et al. [114] for the AVE of the converged validity dimension. The discriminatory validity reflects the empirical differences of all the constructs [115], demonstrating that the constructs are distinguishable, and it requires that the square root of the average variance extracted of the dimensions is higher than the correlation coefficients between the construct and other ones [113]. Table 3 shows that the correlation coefficients between the constructs are lower than the square root of the average variance extracted, indicating that the discriminatory validity is good. To sum up, with good reliability and validity, the measurement model is suitable for analyzing the structural model.

**Table 3:** Square root value between correlation coefficient and AVE of dimensions.

	M	SD	ATT	BI	PV	PEOU	PE	PT	PU
**ATT**	4.813	0.893	**0.812**						
**BI**	4.767	1.236	0.606	**0.889**					
**PV**	5.307	0.975	0.234	0.266	**0.829**				
**PEOU**	4.608	0.923	0.551	0.510	0.156	**0.805**			
**PE**	4.934	1.104	0.507	0.521	0.334	0.476	**0.923**		
**PT**	4.208	1.044	0.498	0.536	0.246	0.518	0.513	**0.917**	
**PU**	4.720	1.041	0.591	0.531	0.137	0.581	0.442	0.525	**0.818**

Note: M = mean;SD = Standard Deviation;The diagonal line is the square root value of the AVE

### Verification of hypotheses

**Fig 2:**
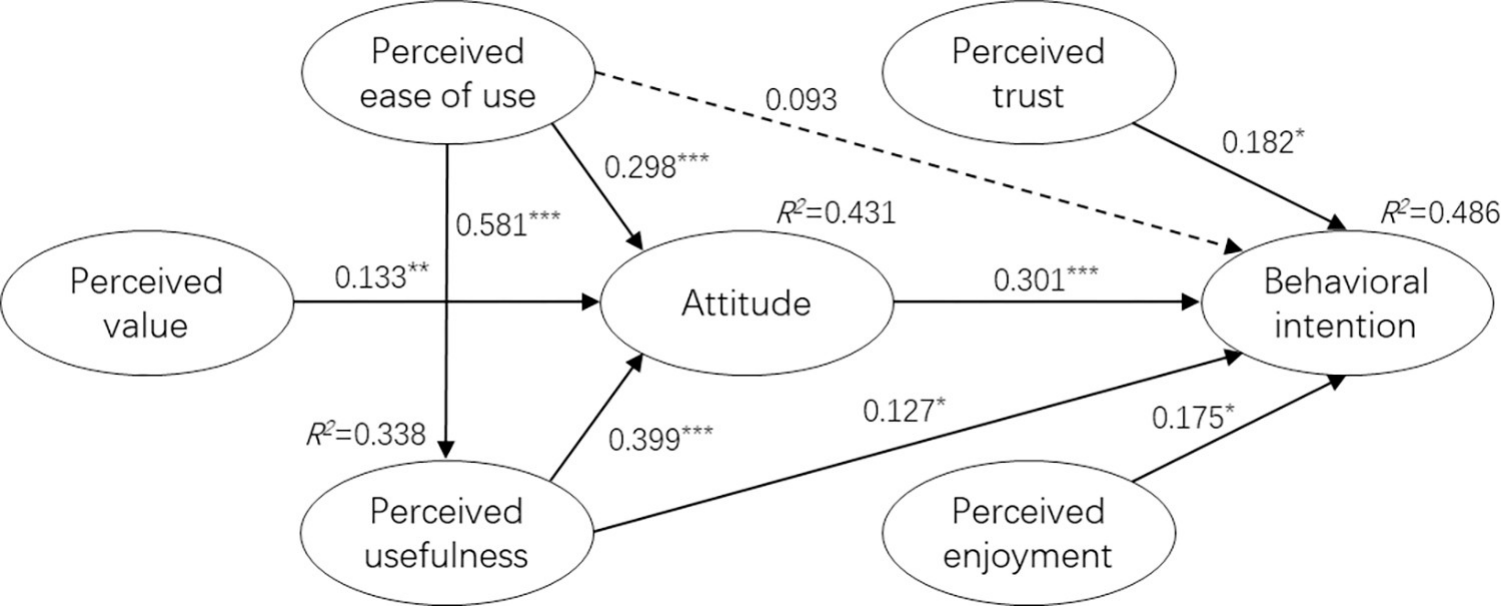
Verification results. Note: *:p<0.05; **: p<0.01; ***: p<0.001.

The significance level of the path coefficient in the structural model is calculated with the 5,000-times Bootstrap resampling method [116], and the results are shown in Fig 2 and Table 4: Perceived usefulness (β = 0.399, t = 5.193; p < 0.001) and perceived ease of use (β = 0.298, t = 4.027; p < 0.001) had a significant effect on attitude, and attitude (β = 0.301, t = 3.593; p < 0.001) has a significant impact on intention to use, so Hypotheses 1, 2, and 4 are valid. Perceived value (β = 0.133, t = 2.387; p < 0.01) also has a significant effect on attitude, which in turn affects intention to use, Hypothesis 9 holds. The study also showed that perceived usefulness (β = 0.127, t = 2.063; p < 0.05) had a significant effect on intention to use. Perceived ease of use (β = 0.581, t = 11.417; p < 0.001) also has a significant impact on perceived usefulness, so Hypotheses 3 and 5 are valid. In addition, participants’ perceived trust (β = 0.182, t = 2.573; p < 0.01) and perceived enjoyment (β = 0.175, t = 2.474; p < 0.05) have a significant impact on the use intention of driverless cars, so Hypotheses 7 and 8 are established. However, our results show that the effect of perceived ease of use on intention is not significant (β = 0.093, t = 1.247), so H6 does not hold. Through the evaluation, it is found that the variables that directly affect the user’s self-driving car use intention from large to small are attitude, perceived trust, perceived enjoyment and perceived usefulness.

**Table 4:** Research hypothesis evaluation results.

Code	Research hypothesis	Path Coefficients	Standard Deviation	t-value	Result
H1	ATT→BI	0.301	0.084	3.593***	Pass the verification
H2	PU→ATT	0.399	0.077	5.193***	Pass the verification
H3	PU→BI	0.127	0.061	2.063*	Pass the verification
H4	PEOU→ATT	0.298	0.074	4.027***	Pass the verification
H5	PEOU→PU	0.581	0.051	11.417***	Pass the verification
H6	PEOU→BI	0.093	0.074	1.247	Verification failed
H7	PT→BI	0.182	0.071	2.573*	Pass the verification
H8	PE→BI	0.175	0.071	2.474*	Pass the verification
H9	PV→ATT	0.133	0.056	2.387**	Pass the verification

Note

*:*p*<0.05

**: *p*<0.01

***: *p*<0.001

### Quality of model

The quality of the model is assessed by the explanatory power, predictive relevance and fit of the model. The calculations of the explanatory power (*R*^2^) of the dependent variable to the self-variant in the structural model are shown as follows: 0.431, 0.486 and 0.338 for attitude, use intention and perceived usefulness respectively, meeting the suggestion proposed by Falk et al. [117] that the independent explanatory power can be determined as qualified if *R*^2^ is higher than 10%. Therefore, *R*^2^ of this study was up to par. The empirical results of this research integrating the three psychological factors of perceived trust, perceived enjoyment, and perceived value into TAM show that the constructed model has good explanatory power, that is, the overall explanatory variation of the empirical research model is 48.6%. This shows that perceived trust, perceived enjoyment, attitude, and perceived usefulness are powerful explanatory factors that affect AVs users’ intentions. Tenenhaus et al. [118] proposed an overall fitness index for the PLS model, namely the Goodness of Fit Index. Goodness of Fit Index is an evaluation index that comprehensively predicts the model after comprehensively considering the measurement model and the structural model. It provides a solution for the model’s global effectiveness fitting evaluation [118]. Calculating the Goodness of Fit Index of this research model is 0.554, reaching the high-fitness threshold value of 0.36 recommended by Hair et al. [108], which shows that the sample data of this research has a high degree of fit with the model. In addition, Hu et al. [119] believed that when the value of Standardized Root Mean Square Residual is less than 0.08, it can be understood that the fit of the model is good. The root of the standardized mean square residual in this study is 0.063, which meets the corresponding standard, so the representative model has a good fit. Henseler et al. [120] believed that when the Q^2^ value of the internal factor facet was higher than 0, it represented that the structural model had predictive relevance to the internal factor facet. In this study, the Q^2^ values for assessing behavioral usage intention, attitude, and perceived usefulness were 0.375, 0.275, and 0.218, respectively, which represented the predictive relevance of the model.

## Discussion

AVs are rapidly developing, but academics’ attention to the studies on acceptance of the emerging technology AVs is insufficient [29]. According to Armitage and Conner [121], in addition to the factor of demographic characteristics, the factor of psychology, as one of the important factors, can promote the reforms in the relevant fields. After all, classic Psychosocial models including Theory of Planned Behavior have been used to explain behaviors related to autonomous driving [34]. And these Psychosocial models may provide relevant information for users’ technology usage intentions [122]. In this study, three psychological factors, perceived trust, perceived value and perceived enjoyment, were included in the technology acceptance model for discussing their role in users’ accepting and using AVs. Research establishes and demonstrates a model for autonomous vehicle acceptance. This study determined the relevant factors affecting potential users’ intention to use AVs, especially the factors relevant to users’ psychological perception. The study found that perceived enjoyment, perceived trust, perceived usefulness, and attitude have a direct positive impact on users’ intention to use. Perceived value, perceived usefulness, and perceived ease of use have a direct positive impact on user attitudes. The results showed that perceived value, perceived enjoyment, perceived trust, perceived usefulness, perceived ease of use, and attitude were all effective predictors of intention to use AVs. This study indicated that in order to enhance users’ intention to use AVs in future, their trust in the vehicles should be built, and these would also be necessary: improving the ease of use and usefulness with regards to man-machine interaction, functions, etc. of AVs, enriching the fun and entertainment experience in AVs, enhancing users’ attitude towards AVs, and emphasizing the characteristics of these dimensions in respect of propaganda.

In the early development stage of AVs, it was considered that users’ perceived trust could affect their behavior under uncertain circumstances [29, 123], and our study demonstrated also this: Perceived trust significantly affects users’ use intention. Our findings are similar to those of Foroughi et al. [16]. This study confirmed the influence of trust, hedonic motivation and other factors on users’ intention to use self-driving cars. Furthermore, our findings on perceived trust are also consistent with those of Meyer-Waarden et al. [124]. Trust may contain various unavoidable risk factors [125], and just as Hegner et al. [20] put it, AVs may increase people’s trust, because there are no human drivers on them, the most common role which driving mistakes come from. Nevertheless, the current users may pay more attention to the AVs themselves in terms of trust in them, such as the ease of use, efficacy, technology, etc. of the product. For instance, according to Lee et al. [126], users may accept use of AVs, when the driving ability of AVs is indeed trustworthy under some complex and unpredictable situations. Of course, the mechanism role between the “risks” and trust in such external roles as drivers deserves our attention. Besides, a study conducted by Payre et al. [127] with AV simulators showed that there is a correlation between trust and practical experience. That is to say, rich practical experience can enhance users’ sense of trust to some extent. However, potential users know little about in what state one drives an automated vehicle [34]. And Hsu ’s research [128] shows that the experience of automation technology owned by users will enhance their sense of adaptability to technology and reduce their fear of technology use. After all, the formation of users’ trust in technology is a dynamic process that gradually increases with the increase of user experience [20]. From this perspective, it is not difficult for us to understand the impact of trust on users’ intention to use technology. Therefore, we should give full play to the positive role of trust in improving users’ intentions to use technology.

As for acceptance of AVs, people may be driven by perceived enjoyment and perceived demand [87, 129]. This study also pointed out that perceived enjoyment significantly affects users’ intention to use AVs. This study result was demonstrated in a previous study [16, 130] as well, namely, it was pointed out that positively perceiving entertainment in automobiles, people will be more willing to use electric automobiles. In the study of Benleulmi et al. [58] on the behavioral use intention of fully autonomous vehicles, it was also confirmed that hedonic factors have a significant impact on the use intention. Enjoyment motivation, an important factor for acceptance and use by users [101], is considered as the foundation on which consumer behavior is understood [95]. It was pointed out that the benefits of enjoyment play an important role in automobile use [131]. According to Eckoldt et al. [132], the psychological distance from the AVs featuring high automaticity and the drivers becomes long, because AVs deprive the user of their driving fun. Therefore, it deserves consideration how to achieve an equilibrium between automation technology level and driving fun and make users accept, for instance, such promotion and guidance as policy can be tried to enhance people’s knowledge about AVs, and at the same time, the entertainment and ease of use in AVs are emphasized, multiple driving modes are provided, etc., so that the user can feel the advantages of and fun brought by AVs. Keszey [57] held that enjoyment type users pursue novel and diversified sensual experience, are willing to bear the corresponding risks, and thus are more willing to accept the novel, exciting and interesting experience as well as the consequent risks brought by AVs [133]. The current young generations like college students, the future potential AV users, have a higher demand for experiencing the fun, fashion, tide and entertainment in technology than seniors, and possibly, their knowledge about automobiles is not limited to a travel tool itself. Thus, in the future R&D design of AVs, the advantages of the Internet of Things, big data and intelligent technology should be brought into fully play for developing the AVs with higher entertainment and fun, in order to provide the potential users with more diversified driving experience and fun, and enhance users’ acceptability.

Perceiving that the benefits obtained through use of a technology are higher than the monetary cost paid for using it, users will regard the perceived value as positive [102], and then their attitude towards the technology will be affected. Research by Yin et al. [48] shows that perceived value has a positive impact on users’ attitudes. Based on field interviews and grounded theory, this study examines the key factors influencing the willingness to purchase electric vehicles for household use among visitors to the 13th Auto Expo in Hainan Province, China, using qualitative analysis methods. The results show that factors such as perceived risk, perceived value and self-control have an impact on users’ purchase intention, among which, perceived value indirectly affects users’ purchase intention through the direct impact on users’ purchase attitude. Our study also found that perceived value significantly affects users’ attitude to use AVs, and that the participants may hold that AVs should have some value, including meeting their basic travel demand and bringing enjoyment to them in travel. So, in the follow-up R&D design of AVs, more attention should be paid to this: Research and design are carried out by centering on users and always on their demands, in order to meet their appeals, and further enhance their recognition of the value of AV products. In addition, our study found that perceived ease of use is not significant for user intention to use AVs, which is consistent with the findings of Park et al. [14] and Yan et al. [80]. However, according to a previous study [34, 134], the relationship between PEOU and use intention is weak, possibly because the driving simulator used in the study [34] impeded the participants’ knowledge to a degree, the participants might be unfamiliar with the operation, etc. of the driving simulating, and accordingly, the study result was affected. However, by taking certain measures to improve the usability and usability of the product, it will bring users higher value and have certain effect on the acceptance and use of users. For instance, the intelligent man-machine interactive control over AVs can be achieved through sounds, tactile sense, thought, etc., and an interface complying with users’ aesthetics and operating habits is designed to enhance their intention use AVs. Of course, the positive impact of perceived usefulness on attitude and intention to use has also been confirmed in our research. This result is consistent with both the early technology acceptance model [70] and recent research [74].

Through this study, we can know that in addition to perceived usefulness, perceived ease of use and attitude in TAM, perceived enjoyment, perceived trust and perceived value are also extremely important factors affecting potential users’ AVs usage intention. Therefore, in order to follow up the development and design of high-acceptance AVs and the promotion of various potential users in society such as college students, whether it is AVs companies or R&D designers, or government agencies and industry organizations that formulate AVs policies, they should focus on The characteristics and needs of potential users, the design, policy formulation, and marketing promotion of AVs are carried out, and the entertainment perception, functional needs, and value pursuits of potential users are incorporated into them, so as to enhance users’ intention to use AVs.

Our research has some theoretical significance. First, although the technology acceptance model is an excellent technology acceptance prediction model, it does not include some other significant belief variables [38], and its application needs to be extended to the environment of autonomous vehicles. In this study, acceptance by users was studied in terms of the emerging technology AVs, and such constructs as perceived trust, perceived value, perceived enjoyment, etc. were added based on the technology acceptance model theory, further expanding the technology acceptance model theory and its scope of application. Second, this study constructs and demonstrates the acceptance model of unmanned vehicles, which can be used for user acceptance research of unmanned vehicles and enriches the user research theory of unmanned vehicles. Third, This study focuses on potential users such as college students, which is different from the current research on tourists [17, 18] and drivers with driving experience [34, 62]. Current college students are potential users of future vehicles, and understanding the factors influencing their use may contribute to the broader commercialization of subsequent AVs. Therefore, it can be said that this study expands the user base for theoretical applications. Fourth, different from the demographic statistics factor in the previous studies [14], discussion was conducted based on users’ psychological factors in the current study, which can enrich the learning about the factors affecting the intention to use AVs. In addition, our research also has some implications for practical applications. First, this study reveals the psychological factors that affect potential users’ intention to accept the use of driverless cars, including perceived trust and perceived enjoyment. These findings could provide direction for driverless car designers or manufacturers to develop features and other features. For example, this study found that perceived enjoyment also has a significant impact on potential users’ intentions to use driverless cars. Therefore, designers of driverless cars should enrich the entertainment function or experience in the process of automotive products and services. Second, the research results can also provide guidance for the marketing and promotion policies of self-driving cars by enterprises or governments. For example, based on the findings of the direct impact of perceived value on users’ attitudes and the indirect impact of use intention in this study, the government or enterprises and other institutions can make users feel the value of driverless cars by clarifying the various benefits brought by the use of driverless cars to society, individuals and families in the development policies and marketing advertisements of the driverless car industry. Understand the usefulness of driverless cars, increase the positive attitude of users, and ultimately improve their intention to use them. In conclusion, this study provides predictors of AV acceptance for AV designers, automakers, automotive policy makers, and related practitioners. This helps them make actionable autonomous vehicle-related decisions to promote high-acceptance autonomous vehicle research and development and increase users’ intention to use autonomous vehicles.

There are some limitations to this study. First, no true AVs were provided for the participants for experiencing in this study. Yet the tests in which no study products are used are still of some value to the studies on potential users’ intention to use the AVs that are still in the early development stage. Of course, in the follow-up studies, we can try to provide driving simulators, etc. just as Buckley et al. [34] did, in order to obtain richer study results. Second, the user’s car driving experience may have a certain impact on the user’s behavior, and the experience will be enriched with time, after all, time may have an impact on the user’s behavior [34]. Respondents in this study included some potential users with no driving experience, which may have some influence on the findings. So, cross-section exploration was insufficient in this study. A longitudinal study lasting for a longer period of time should be carried out in the future. Even, independent targeted studies of users with or without experience with self-driving cars might yield some interesting findings. In addition, the factors influencing the acceptance and use of AVs are complex, and this study is limited to focus on individual psychological factors. In the future studies, it should be tried to include more psychological factors based on the results of this study, so as to more deeply discuss potential users’ intention to use AVs.

## Conclusions

In recent years, driverless cars have attracted much attention. To this end, there is an urgent need to understand the state of user acceptance of drone-driven vehicles. However, although the research on user intention of self-driving cars has gradually attracted attention, only a few previous studies have focused on psychological factors [35, 40, 41]. This study incorporates three psychological factors of perceived trust, perceived value and perceived enjoyment into the technology acceptance model. This study disclosed the causes for users’ intention to use AVs and expanded people’s understanding about the intention to use AVs. In this study, the relationships between perceived trust, perceived value, perceived enjoyment, perceived usefulness, perceived ease of use, attitude and use intention were analysed in the structural equation mode. It was found that perceived enjoyment, perceived trust, perceived usefulness, and attitude had a direct positive impact on users’ intention to use. Perceived value, perceived usefulness, and perceived ease of use have a direct positive impact on user attitudes. In addition, perceived ease of use has also been shown to directly affect perceived usefulness. The results showed that perceived value, perceived enjoyment, perceived trust, perceived usefulness, perceived ease of use and attitude can be used to predict and explain users’ acceptance and use of AVs. The factors affecting users’ intention to use AVs can be more comprehensively understood, through an analysis on the psychological factors in the theory, which provides a reference for the development and design of driverless cars. For example, based on the results of this study, designers can focus on practicality, operability, entertainment experience, and human-computer interaction when conducting functional design and technical research of driverless cars. Therefore, it can be said that this study enriches the learning by AVs designers about the factors affecting users’ intention to use AVs, and can be used as reference by AVs designers and marketers, in order to promote the R&D design of AVs featuring high acceptability and the users’ intention to use AVs. At the same time, this study further expands the science and technology acceptance model theory and its applicable fields. The research constructs and demonstrates the acceptance model of unmanned vehicles, which can be used for user acceptance research of unmanned vehicles and enriches the user research theory of unmanned vehicles.

## Supporting information

S1 File (RAR)

## References

[1] Anderson JM, Nidhi K, Stanley KD, Sorensen P, Samaras C, Oluwatola OA. Autonomous vehicle technology: A guide for policymakers: Rand Corporation; 2014.

[2] Kuo P-H, Krishnamurthy A, Malmborg CJ. Design models for unit load storage and retrieval systems using autonomous vehicle technology and resource conserving storage and dwell point policies. Applied Mathematical Modelling. 2007;31(10):2332–46.

[3] Alessandrini A, Cattivera A, Holguin C, Stam D. CityMobil2: challenges and opportunities of fully automated mobility. Road vehicle automation. 2014 169–84.

[4] Hevelke A, Nida-Rümelin J. Responsibility for crashes of autonomous vehicles: An ethical analysis. Sci Eng Ethics. 2015;21(3):619–30. doi: 10.1007/s11948-014-9565-5 25027859 PMC4430591

[5] Times G. Beijing launches China’s first commercial trial of autonomous driving services. Global Times; 2021.https://www.globaltimes.cn/page/202111/1239891.shtml.accessed 12.08.2022.

[6] Xu X, Fan C-K. Autonomous vehicles, risk perceptions and insurance demand: An individual survey in China. Transportation research part A: policy and practice. 2019;124549–56.

[7] Poliak M, Poliakova A, Zhuravleva NA, Nica E. Identifying the Impact of Parking Policy on Road Transport Economics. Mobile Networks and Applications. 2021 1–8.

[8] Poliak M, Poliakova A, Svabova L, Zhuravleva NA, Nica E. Competitiveness of price in international road freight transport. Journal of Competitiveness. 2021;13(2):83–98.

[9] Konecny V, Jaskiewicz M, Downs S. Motion Planning and Object Recognition Algorithms, Vehicle Navigation and Collision Avoidance Technologies, and Geospatial Data Visualization in Network Connectivity Systems. Contemp Readings L & Soc Just. 2022;1489.

[10] Kliestik T, Musa H, Machova V, Rice LF. Remote Sensing Data Fusion Techniques, Autonomous Vehicle Driving Perception Algorithms, and Mobility Simulation Tools in Smart Transportation System. Contemp Readings L & Soc Just. 2022;14137.

[11] Guo Y, Souders D, Labi S, Peeta S, Benedyk I, Li Y. Paving the way for autonomous Vehicles: Understanding autonomous vehicle adoption and vehicle fuel choice under user heterogeneity. Transportation Research Part A: Policy and Practice. 2021;154364–98.

[12] Litman T. Autonomous vehicle implementation predictions: Implications for transport planning. 2020.

[13] Placek M. Worldwide–Driverless Car Market Size 2025. Statista; 2021.https://www.statista.com/statistics/1224515/av-market-size-worldwide-forecast/. accessed 12.08.2022.

[14] Park J, Hong E, Le H. Adopting autonomous vehicles: The moderating effects of demographic variables. Journal of Retailing and Consumer Services. 2021;63.

[15] Joo YK, Kim B. Selfish but Socially Approved: The Effects of Perceived Collision Algorithms and Social Approval on Attitudes toward Autonomous Vehicles. Int J Hum-Comput Interact. 2022.

[16] Foroughi B, Nhan PV, Iranmanesh M, Ghobakhloo M, Nilashi M, Yadegaridehkordi E. Determinants of intention to use autonomous vehicles: Findings from PLS-SEM and ANFIS. Journal of Retailing and Consumer Services. 2023;70.

[17] Ribeiro MA, Gursoy D, Chi OH. Customer Acceptance of Autonomous Vehicles in Travel and Tourism. Journal of Travel Research. 2022;61(3):620–36.

[18] Jászberényi M, Miskolczi M, Munkácsy A, Földes D. What drives tourists to adopt self-driving cars? Transportation research part F: traffic psychology and behaviour. 2022;89407–22.

[19] Prideaux B, Yin P. The disruptive potential of autonomous vehicles (AVs) on future low-carbon tourism mobility. Asia Pac J Tour Res. 2019;24(5):459–67.

[20] Hegner SM, Beldad AD, Brunswick GJ. In automatic we trust: investigating the impact of trust, control, personality characteristics, and extrinsic and intrinsic motivations on the acceptance of autonomous vehicles. International Journal of Human–Computer Interaction. 2019;35(19):1769–80.

[21] Fagnant DJ, Kockelman K. Preparing a nation for autonomous vehicles: opportunities, barriers and policy recommendations. Transportation Research Part A: Policy and Practice. 2015;77167–81.

[22] Ferreras LE. The driverless city. Civil Engineering Magazine Archive. 2014;84(3):52–5.

[23] Waung M, McAuslan P, Lakshmanan S. Trust and intention to use autonomous vehicles: Manufacturer focus and passenger control. Transportation research part F: traffic psychology and behaviour. 2021;80328–40.

[24] Milakis D, Van Arem B, Van Wee B. Policy and society related implications of automated driving: A review of literature and directions for future research. Journal of Intelligent Transportation Systems. 2017;21(4):324–48.

[25] Faisal A, Kamruzzaman M, Yigitcanlar T, Currie G. Understanding autonomous vehicles. Journal of transport and land use. 2019;12(1):45–72.

[26] Golbabaei F, Yigitcanlar T, Bunker J. The role of shared autonomous vehicle systems in delivering smart urban mobility: A systematic review of the literature. International Journal of Sustainable Transportation. 2020 1–18.

[27] Rubio F, Llopis-Albert C, Valero F, Besa AJ. Sustainability and optimization in the automotive sector for adaptation to government vehicle pollutant emission regulations. Journal of Business Research. 2020;112561–6.

[28] Menon N, Pinjari A, Zhang Y, Zou L. Consumer perception and intended adoption of autonomous-vehicle technology: Findings from a university population survey. In Proceedings of the Transportation Research Board 95th Annual Meeting. Washington, DC, USA 2016.

[29] Zhang T, Tao D, Qu X, Zhang X, Zeng J, Zhu H, et al. Automated vehicle acceptance in China: Social influence and initial trust are key determinants. Transportation research part C: emerging technologies. 2020;112220–33.

[30] Zhang T, Tao D, Qu X, Zhang X, Lin R, Zhang W. The roles of initial trust and perceived risk in public’s acceptance of automated vehicles. Transportation research part C: emerging technologies. 2019;98207–20.

[31] Zoellick JC, Kuhlmey A, Schenk L, Schindel D, Blüher S. Assessing acceptance of electric automated vehicles after exposure in a realistic traffic environment. PLoS One. 2019;14(5):e0215969. doi: 10.1371/journal.pone.0215969 31048877 PMC6497263

[32] Wu ZY, Zhou HM, Xi HJ, Wu N. Analysing public acceptance of autonomous buses based on an extended TAM model. Iet Intelligent Transport Systems. 2021;15(10):1318–30.

[33] Lee J, Lee D, Park Y, Lee S, Ha T. Autonomous vehicles can be shared, but a feeling of ownership is important: Examination of the influential factors for intention to use autonomous vehicles. Transportation Research Part C: Emerging Technologies. 2019;107411–22.

[34] Buckley L, Kaye S-A, Pradhan AK. Psychosocial factors associated with intended use of automated vehicles: A simulated driving study. Accident Analysis & Prevention. 2018;115202–8. doi: 10.1016/j.aap.2018.03.021 29631216

[35] Choi JK, Ji YG. Investigating the importance of trust on adopting an autonomous vehicle. Int J Hum-Comput Interact. 2015;31(10):692–702.

[36] Alalwan AA, Baabdullah AM, Rana NP, Tamilmani K, Dwivedi YK. Examining adoption of mobile internet in Saudi Arabia: Extending TAM with perceived enjoyment, innovativeness and trust. Technol Soc. 2018;55100–10.

[37] Beldad AD, Hegner SM. Expanding the technology acceptance model with the inclusion of trust, social influence, and health valuation to determine the predictors of German users’ willingness to continue using a fitness app: A structural equation modeling approach. International Journal of Human–Computer Interaction. 2018;34(9):882–93.

[38] Benbasat I, Barki H. Quo vadis TAM? Journal of the association for information systems. 2007;8(4):211–8.

[39] Tian YQ, Wang XW. A study on psychological determinants of users’ autonomous vehicles adoption from anthropomorphism and UTAUT perspectives. Front Psychol. 2022;13. 10.3389/fpsyg.2022.986800 PMC9426542 36051203

[40] Jing P, Huang H, Ran B, Zhan F, Shi Y. Exploring the factors affecting mode choice Intention of autonomous vehicle based on an extended theory of planned behavior—A case study in China. Sustainability. 2019;11(4):1155.

[41] Verberne FM, Ham J, Midden CJ. Trust in smart systems: Sharing driving goals and giving information to increase trustworthiness and acceptability of smart systems in cars. Hum Factors. 2012;54(5):799–810. doi: 10.1177/0018720812443825 23156624

[42] Molnar LJ, Ryan LH, Pradhan AK, Eby DW, Louis RMS, Zakrajsek JS. Understanding trust and acceptance of automated vehicles: An exploratory simulator study of transfer of control between automated and manual driving. Transportation research part F: traffic psychology and behaviour. 2018;58319–28.

[43] Chen H-K, Yan D-W. Interrelationships between influential factors and behavioral intention with regard to autonomous vehicles. International journal of sustainable transportation. 2019;13(7):511–27.

[44] Liu H, Yang R, Wang L, Liu P. Evaluating initial public acceptance of highly and fully autonomous vehicles. International Journal of Human–Computer Interaction. 2019;35(11):919–31.

[45] Zeithaml VA. Consumer perceptions of price, quality, and value: a means-end model and synthesis of evidence. Journal of marketing. 1988;52(3):2–22.

[46] Li Z, Yuan J, Du B, Hu J, Yuan W, Palladini L, et al. Customer Behavior on Purchasing Channels of Sustainable Customized Garment With Perceived Value and Product Involvement. Front Psychol. 2020;113581. doi: 10.3389/fpsyg.2020.588512 33408664 PMC7779488

[47] Ström R, Vendel M, Bredican J. Mobile marketing: A literature review on its value for consumers and retailers. Journal of Retailing and Consumer Services. 2014;21(6):1001–12.

[48] Yin YR, Li Y, Zhang Y. Influencing factor analysis of household electric vehicle purchase intention of HaiNan Free Trade Port under the background of low-carbon lifestyle. Energy Reports. 2022;8569–79.

[49] Steg L. Car use: lust and must. Instrumental, symbolic and affective motives for car use. Transportation Research Part A: Policy and Practice. 2005;39(2–3):147–62.

[50] Calder BJ, Staw BM. Self-perception of intrinsic and extrinsic motivation. Journal of personality and social psychology. 1975;31(4):599. doi: 10.1037/h0077100 1159610

[51] Scott W Jr, Farh J-L, Podsakoff PM. The effects of “intrinsic” and “extrinsic” reinforcement contingencies on task behavior. Organizational Behavior and Human Decision Processes. 1988;41(3):405–25.

[52] Davis FD, Bagozzi RP, Warshaw PR. Extrinsic and intrinsic motivation to use computers in the workplace 1. Journal of applied social psychology. 1992;22(14):1111–32.

[53] Becker F, Axhausen KW. Literature review on surveys investigating the acceptance of automated vehicles. Transportation. 2017;44(6):1293–306.

[54] Venkatesh V, Thong JY, Xu X. Consumer acceptance and use of information technology: extending the unified theory of acceptance and use of technology. MIS quarterly. 2012157–78.

[55] Holbrook MB, Hirschman EC. The experiential aspects of consumption: Consumer fantasies, feelings, and fun. Journal of consumer research. 1982;9(2):132–40.

[56] Haboucha CJ, Ishaq R, Shiftan Y. User preferences regarding autonomous vehicles. Transportation Research Part C: Emerging Technologies. 2017;7837–49.

[57] Keszey T. Behavioural intention to use autonomous vehicles: Systematic review and empirical extension. Transportation research part C: emerging technologies. 2020;119102732.

[58] Benleulmi AZ, Ramdani B. Behavioural intention to use fully autonomous vehicles: Instrumental, symbolic, and affective motives. Transportation Research Part F-Traffic Psychology and Behaviour. 2022;86226–37.

[59] Talebian A, Mishra S. Predicting the adoption of connected autonomous vehicles: A new approach based on the theory of diffusion of innovations. Transportation Research Part C: Emerging Technologies. 2018;95363–80.

[60] Xu Z, Zhang K, Min H, Wang Z, Zhao X, Liu P. What drives people to accept automated vehicles? Findings from a field experiment. Transportation Research Part C: Emerging Technologies. 2018;95320–34.

[61] Charness N, Yoon JS, Souders D, Stothart C, Yehnert C. Predictors of Attitudes Toward Autonomous Vehicles: The Roles of Age, Gender, Prior Knowledge, and Personality. Front Psychol. 2018;9. doi: 10.3389/fpsyg.2018.02589 30631296 PMC6315114

[62] Useche SA, Penaranda-Ortega M, Gonzalez-Marin A, Llamazares FJ. Assessing the Effect of Drivers’ Gender on Their Intention to Use Fully Automated Vehicles. Appl Sci-Basel. 2022;12(1).

[63] Huang TY. Research on the use intention of potential designers of unmanned cars based on technology acceptance model. PLoS One. 2021;16(8). doi: 10.1371/journal.pone.0256570 34415950 PMC8378682

[64] Davis FD. Perceived usefulness, perceived ease of use, and user acceptance of information technology. MIS quarterly. 1989 319–40.

[65] Madigan R, Louw T, Dziennus M, Graindorge T, Ortega E, Graindorge M, et al. Acceptance of automated road transport systems (ARTS): an adaptation of the UTAUT model. Transportation Research Procedia. 2016;142217–26.

[66] Yousafzai SY, Foxall GR, Pallister JG. Technology acceptance: a meta‐analysis of the TAM: Part 1. Journal of Modelling in Management. 2007.

[67] Kaur K, Rampersad G. Trust in driverless cars: Investigating key factors influencing the adoption of driverless cars. J Eng Technol Manage. 2018;4887–96.

[68] Zhang T, Chan AH, Li S, Zhang W, Qu X. Driving anger and its relationship with aggressive driving among Chinese drivers. Transportation research part F: traffic psychology and behaviour. 2018;56496–507.

[69] Venkatesh V, Morris MG, Davis GB, Davis FD. User acceptance of information technology: Toward a unified view. Mis Quarterly. 2003;27(3):425–78.

[70] Davis FD, Bagozzi RP, Warshaw PR. User acceptance of computer technology: a comparison of two theoretical models. Management science. 1989;35(8):982–1003.

[71] Holden RJ, Karsh BT. The Technology Acceptance Model: Its past and its future in health care. J Biomed Inform. 2010;43(1):159–72. doi: 10.1016/j.jbi.2009.07.002 19615467 PMC2814963

[72] Payre W, Cestac J, Delhomme P. Intention to use a fully automated car: Attitudes and a priori acceptability. Transportation research part F: traffic psychology and behaviour. 2014;27252–63.

[73] Petschnig M, Heidenreich S, Spieth P. Innovative alternatives take action–Investigating determinants of alternative fuel vehicle adoption. Transportation Research Part A: Policy and Practice. 2014;6168–83.

[74] Wang N, Pei Y, Fu H. Public Acceptance of Last-Mile Shuttle Bus Services with Automation and Electrification in Cold-Climate Environments. Sustainability. 2022;14(21):14383.

[75] Wang NH, Pei YL, Wang YJ. Antecedents in Determining Users’ Acceptance of Electric Shuttle Bus Services. Mathematics. 2022;10(16).

[76] Suresh P, Manivannan P. Reduction of vehicular pollution through fuel economy improvement with the use of autonomous self-driving passenger cars. Journal of Environmental Research And Development. 2014;8(3A):705.

[77] Koul S, Eydgahi A. Utilizing technology acceptance model (TAM) for driverless car technology adoption. Journal of technology management & innovation. 2018;13(4):37–46.

[78] Stiegemeier D, Kraus J, Bringeland S, Baumann M. Motivated to Use: Beliefs and Motivation Influencing the Acceptance and Use of Assistance and Navigation Systems. Int J Hum-Comput Interact.

[79] Li G, Liang YK, Wang HQ, Chen JL, Chang XB. Factors Influencing Users’ Willingness to Adopt Connected and Autonomous Vehicles: Net and Configurational Effects Analysis Using PLS-SEM and FsQCA. Journal of Advanced Transportation. 2022;2022.

[80] Yan YY, Zhong SQ, Tian JF, Li T. Continuance intention of autonomous buses: An empirical analysis based on passenger experience. Transport Policy. 2022;12685–95.

[81] Müller JM. Comparing technology acceptance for autonomous vehicles, battery electric vehicles, and car sharing—A study across Europe, China, and North America. Sustainability. 2019;11(16):4333.

[82] Setiawan M, Setyawati CY. The influence of perceived ease of use on the intention to use mobile payment. Journal of Accounting and Strategic Finance. 2020;3(1):18–32.

[83] Salloum SA, Alhamad AQM, Al-Emran M, Monem AA, Shaalan K. Exploring students’ acceptance of e-learning through the development of a comprehensive technology acceptance model. IEEE Access. 2019;7128445–62.

[84] Jing C, Liu XD, Li K, Liu X, Dong B, Dong F, et al. The pseudocapacitance mechanism of graphene/CoAl LDH and its derivatives: Are all the modifications beneficial? Journal of Energy Chemistry. 2021;52218–27.

[85] Wu J, Liao H, Wang J-W, Chen T. The role of environmental concern in the public acceptance of autonomous electric vehicles: A survey from China. Transportation Research Part F: Traffic Psychology and Behaviour. 2019;6037–46.

[86] Lee JD, See KA. Trust in automation: Designing for appropriate reliance. Hum Factors. 2004;46(1):50–80. doi: 10.1518/hfes.46.1.50_30392 15151155

[87] Herrenkind B, Brendel AB, Nastjuk I, Greve M, Kolbe LM. Investigating end-user acceptance of autonomous electric buses to accelerate diffusion. Transportation Research Part D: Transport and Environment. 2019;74255–76.

[88] Pavlou PA. Consumer acceptance of electronic commerce: Integrating trust and risk with the technology acceptance model. International journal of electronic commerce. 2003;7(3):101–34.

[89] Tussyadiah IP, Zach FJ, Wang J. Attitudes toward autonomous on demand mobility system: The case of self-driving taxi. Information and communication technologies in tourism 2017: Springer; 2017. p. 755–66.

[90] Gefen D, Karahanna E, Straub DW. Trust and TAM in online shopping: An integrated model. MIS quarterly. 2003 51–90.

[91] Parasuraman R, Riley V. Humans and automation: Use, misuse, disuse, abuse. Hum Factors. 1997;39(2):230–53.

[92] Jing P, Xu G, Chen Y, Shi Y, Zhan F. The determinants behind the acceptance of autonomous vehicles: A systematic review. Sustainability. 2020;12(5):1719.

[93] Cho Y, Park J, Park S, Jung ES. Technology acceptance modeling based on user experience for autonomous vehicles. Journal of the Ergonomics Society of Korea. 2017;36(2):87–108.

[94] Zhang T, Zeng W, Zhang Y, Tao D, Li G, Qu X. What drives people to use automated vehicles? A meta-analytic review. Accident Analysis & Prevention. 2021;159106270. doi: 10.1016/j.aap.2021.106270 34216854

[95] Childers TL, Carr CL, Peck J, Carson S. Hedonic and utilitarian motivations for online retail shopping behavior. Journal of retailing. 2001;77(4):511–35.

[96] Van der Heijden HJMq. User acceptance of hedonic information systems. 2004 695–704.

[97] Venkatesh V. Determinants of perceived ease of use: Integrating perceived behavioral control, computer anxiety and enjoyment into the technology acceptance model. Inf Syst Res. 2000;11(4):342–65.

[98] Alalwan AA, Dwivedi YK, Rana NP. Factors influencing adoption of mobile banking by Jordanian bank customers: Extending UTAUT2 with trust. International Journal of Information Management. 2017;37(3):99–110.

[99] Huang C-Y, Kao Y-S. UTAUT2 based predictions of factors influencing the technology acceptance of phablets by DNP. Math Probl Eng. 2015;20151–23.

[100] Gkartzonikas C, Gkritza K. What have we learned? A review of stated preference and choice studies on autonomous vehicles. Transportation Research Part C: Emerging Technologies. 2019;98323–37.

[101] Madigan R, Louw T, Wilbrink M, Schieben A, Merat N. What influences the decision to use automated public transport? Using UTAUT to understand public acceptance of automated road transport systems. Transportation research part F: traffic psychology and behaviour. 2017;5055–64.

[102] Yuen KF, Huyen DTK, Wang X, Qi G. Factors influencing the adoption of shared autonomous vehicles. Int J Environ Res Public Health. 2020;17(13):4868. doi: 10.3390/ijerph17134868 32640662 PMC7369749

[103] M J H.K.D.H., Hewapathirana NT, Dk T. Impact of Perceived Value on Customer Adoption: Examining the Moderating Effect of Perceived Risk TowardsAlternative Fuel Vehicles. International Journal of Business and Management Invention. 2022;11(9):75–92.

[104] Vinijcharoensri K. Luxury value perceptions and attitude toward purchase intention of luxury automobiles in Thailand. HUMAN BEHAVIOR, DEVELOPMENT and SOCIETY. 2016;13(2):46–56.

[105] Bennett R, Vijaygopal R, Kottasz R. Willingness of people who are blind to accept autonomous vehicles: An empirical investigation. Transportation research part F: traffic psychology and behaviour. 2020;6913–27.

[106] Nordhoff S, Louw T, Innamaa S, Lehtonen E, Beuster A, Torrao G, et al. Using the UTAUT2 model to explain public acceptance of conditionally automated (L3) cars: A questionnaire study among 9,118 car drivers from eight European countries. Transportation research part F: traffic psychology and behaviour. 2020;74280–97.

[107] Cheung CM, Lee MK. What drives consumers to spread electronic word of mouth in online consumer-opinion platforms. Decision support systems. 2012;53(1):218–25.

[108] Hair JF, Ringle CM, Sarstedt M. PLS-SEM: Indeed a silver bullet. Journal of Marketing theory and Practice. 2011;19(2):139–52.

[109] Bollen KA, Stine RA. Bootstrapping goodness-of-fit measures in structural equation models. Sociological Methods & Research. 1992;21(2):205–29.

[110] Wang Y-Y, Wang Y-S, Lin T-C. Developing and validating a technology upgrade model. International Journal of Information Management. 2018;38(1):7–26.

[111] Podsakoff PM, Organ DW. Self-reports in organizational research: Problems and prospects. Journal of management. 1986;12(4):531–44.

[112] Hair J, Anderson R, Tatham R, Black W. Multivariate Data Analysis, 5th edPrentice-Hall. Englewood Cliffs, NJ. 1998.

[113] Fornell C, Larcker DF. Evaluating structural equation models with unobservable variables and measurement error. Journal of marketing research. 1981;18(1):39–50.

[114] Bagozzi RP, Yi Y. On the evaluation of structural equation models. Journal of the academy of marketing science. 1988;16(1):74–94.

[115] Ab Hamid M, Sami W, Sidek MM. Discriminant validity assessment: Use of Fornell & Larcker criterion versus HTMT criterion. Journal of Physics: Conference Series: IOP Publishing; 2017. p. 012163.

[116] Hair JF, Sarstedt M, Ringle CM, Mena JA. An assessment of the use of partial least squares structural equation modeling in marketing research. Journal of the academy of marketing science. 2012;40(3):414–33.

[117] Falk RF, Miller NB. A primer for soft modeling. Akron: University of Akron Press; 1992.

[118] Tenenhaus M, Vinzi VE, Chatelin Y-M, Lauro C. PLS path modeling. Computational statistics & data analysis. 2005;48(1):159–205.

[119] Hu LT, Bentler PM. Fit indices in covariance structure modeling: Sensitivity to underparameterized model misspecification. Psychological Methods. 1998;3(4):424–53.

[120] Henseler J, Ringle CM, Sinkovics RR. The use of partial least squares path modeling in international marketing. Advances in International Marketing. 2009;20277–320.

[121] Armitage CJ, Conner M. Efficacy of the theory of planned behaviour: A meta‐analytic review. British journal of social psychology. 2001;40(4):471–99. doi: 10.1348/014466601164939 11795063

[122] Kyriakidis M, Happee R, de Winter JC. Public opinion on automated driving: Results of an international questionnaire among 5000 respondents. Transportation research part F: traffic psychology and behaviour. 2015;32127–40.

[123] Liu P, Guo Q, Ren F, Wang L, Xu Z. Willingness to pay for self-driving vehicles: Influences of demographic and psychological factors. Transportation Research Part C: Emerging Technologies. 2019;100306–17.

[124] Meyer-Waarden L, Cloarec J. “Baby, you can drive my car”: Psychological antecedents that drive consumers’ adoption of AI-powered autonomous vehicles. Technovation. 2022;109102348.

[125] Weigert A, Lewis D. Trust as a social reality. Social Forces. 1985;63(4):967–85.

[126] Lee J-G, Kim KJ, Lee S, Shin D-H. Can autonomous vehicles be safe and trustworthy? Effects of appearance and autonomy of unmanned driving systems. Int J Hum-Comput Interact. 2015;31(10):682–91.

[127] Payre W, Cestac J, Delhomme P. Fully automated driving: Impact of trust and practice on manual control recovery. Hum Factors. 2016;58(2):229–41. doi: 10.1177/0018720815612319 26646299

[128] Hsu J. 75% of US drivers fear self-driving cars, but it’s an easy fear to get over. IEEE Spectrum, March. 2016;72016.

[129] Kapser S, Abdelrahman M. Acceptance of autonomous delivery vehicles for last-mile delivery in Germany–Extending UTAUT2 with risk perceptions. Transportation Research Part C: Emerging Technologies. 2020;111210–25.

[130] Schuitema G, Anable J, Skippon S, Kinnear N. The role of instrumental, hedonic and symbolic attributes in the intention to adopt electric vehicles. Transportation Research Part A: Policy and Practice. 2013;4839–49.

[131] Bergstad CJ, Gamble A, Hagman O, Polk M, Gärling T, Olsson LE. Affective–symbolic and instrumental–independence psychological motives mediating effects of socio-demographic variables on daily car use. Journal of Transport Geography. 2011;19(1):33–8.

[132] Eckoldt K, Knobel M, Hassenzahl M, Schumann J. An experiential perspective on advanced driver assistance systems. It-Information Technology. 2012;54(4):165–71.

[133] Osswald S, Wurhofer D, Trösterer S, Beck E, Tscheligi M. Predicting information technology usage in the car: towards a car technology acceptance model. Proceedings of the 4th International Conference on Automotive User Interfaces and Interactive Vehicular Applications2012. p. 51–8.

[134] Rahman MM, Lesch MF, Horrey WJ, Strawderman L. Assessing the utility of TAM, TPB, and UTAUT for advanced driver assistance systems. Accident Analysis & Prevention. 2017;108361–73. doi: 10.1016/j.aap.2017.09.011 28957759

